# SCLpred-ECL: Subcellular Localization Prediction by Deep N-to-1 Convolutional Neural Networks

**DOI:** 10.3390/ijms25105440

**Published:** 2024-05-16

**Authors:** Maryam Gillani, Gianluca Pollastri

**Affiliations:** School of Computer Science, University College Dublin (UCD), D04 V1W8 Dublin, Ireland; gianluca.pollastri@ucd.ie

**Keywords:** protein subcellular localization prediction, N-to-1 Convolutional Neural Networks, deep learning, bioinformatics

## Abstract

The subcellular location of a protein provides valuable insights to bioinformaticians in terms of drug designs and discovery, genomics, and various other aspects of medical research. Experimental methods for protein subcellular localization determination are time-consuming and expensive, whereas computational methods, if accurate, would represent a much more efficient alternative. This article introduces an ab initio protein subcellular localization predictor based on an ensemble of Deep N-to-1 Convolutional Neural Networks. Our predictor is trained and tested on strict redundancy-reduced datasets and achieves 63% accuracy for the diverse number of classes. This predictor is a step towards bridging the gap between a protein sequence and the protein’s function. It can potentially provide information about protein–protein interaction to facilitate drug design and processes like vaccine production that are essential to disease prevention.

## 1. Introduction

Protein subcellular localization prediction is of great significance in bioinformatics and biological research [1]. The knowledge of the subcellular location of a protein provides valuable information about its functionalities, the functioning of the cell, and its possible interactions with other proteins. It helps in determining the environments in which proteins operate. It can aid drug design, identification of vaccine targets, and countless other life-saving applications [2]. Since conventional experimental methods are labor-intensive, costly, and time-consuming, machine learning-based protein subcellular location determination has significantly progressed in the last two decades. Also, with the rapid development of next-generation sequencing technologies, the sheer amount of protein sequences is being discovered continuously. A large amount of annotated sequence data makes experimental methods impractical and expensive [3]. Therefore, there has been a major effort among bioinformaticians to utilize computational methods that could potentially help in dealing with massive sizes of sequence data within reasonable time, cost, and good prediction accuracy [4].

During the past two decades, most of the predictors of protein subcellular localization have been based on machine learning algorithms, from Support Vector Machines (SVM) [5] to Deep Neural Networks [6] and various statistical models such as covariant discriminant algorithms [7] and Bayesian networks [8]. Computational methods for predicting protein subcellular localization can primarily be divided into two categories, i.e., homology-based function prediction or template-based modeling and ab initio prediction of protein function or template-free approaches [5]. Protein subcellular localization is closely related to protein functions. In homology-based function, proteins with similar sequences often have similar functions [6]. If we know the function of one of them, we can infer the function of the other one. On the other hand, ab initio prediction of protein function is where we rely on what is written in the sequence, or homologues of unknown annotation, and on what we can predict about the location, as sequence annotations describe regions or sites of interest in the protein sequence [7].

Predicting a subcellular location of a protein from its amino acid sequence is considered to be a challenging task even after several decades of efforts [8]. However, if the query protein has homologues of known structure, the prediction task becomes relatively easy, and high-resolution models can be produced by simply copying and refining the framework of the solved structure (also known as template-based modelling) [9]. However, when these structural homologues do not exist or are identified particularly, we need to construct the models from scratch. This kind of method is called ab initio prediction and provides a complete solution to the subcellular localization of unannotated protein problem. Currently, the prediction accuracy of ab initio modelling is low and the success is generally limited to a small portion of protein sequences. Despite the considerable recent progress, subcellular localization predictions for proteins that do not have sequence and structure homologues are yet to be improved.

Considering the aforementioned challenges, the proposed research considers a broader range of subcellular localization classes compared to previous studies, which typically focus on a limited number of classes. This expansion of classes enhances the applicability and relevance of your findings across various protein subcellular prediction contexts. Secondly, deeper network layers are incorporated into the prediction model, which is a more complex and comprehensive approach to subcellular localization prediction. This deeper architecture allows for better representation and understanding of detailed protein characteristics, leading to more accurate predictions. Third, the proposed approach achieves higher prediction accuracy compared to existing predictors across a broader set of classes. This improvement signifies the effectiveness and reliability of our methodology in accurately predicting protein subcellular localization across diverse classes.

## 2. Literature Review

Recent advancements in deep learning have fueled the development of various tools and methods in subcellular localization predictions. The research gap related to modelling the accuracy of the proteins that lack sequence and relevant homologues has been addressed recently. Even though many protein subcellular location prediction systems have been introduced recently, modern state-of-the-art solutions based on machine learning algorithms that could support large amounts of protein data in a reasonable time, cost, and good prediction accuracy are still lacking [9]. There are few recent surveys that extensively cover protein subcellular localization prediction tools and methods [1,2,3,9,10,11,12,13]. However, there are very few ab initio predictors available so far; we have covered a few relevant and state-of-the-art tools and methods in this section to cover the relevant literature.

DeepLoc 2.0 [14] and DeepLoc 1.0 [6] are subcellular localization prediction models. DeepLoc 1.0 [6] uses three-stage deep learning methods for sequence classification. During the first stage, sequences from amino acids are generated. Then, the attention-based pooling stage constructs a single representation from the whole sequence followed by a prediction via classifier. On the other hand, DeepLoc 2.0 [14] adapts the same model with slight modifications that include a pre-trained protein transformer language model. The subcellular localization prediction accuracy of DeepLoc 2.0 is better than DeepLoc 1.0. SCLpred-MEM [15] and SCLpred-EMS [16] are SCLpred family of ab initio Subcellular localization predictors based on an ensemble of Deep N-to-1 Convolutional Neural Networks (N1-NNs). SCLpred-MEM works in conjunction with SCLpred-EMS [16] to classify part of the endomembrane system and secretory pathway. The proteins classified by SCLpred-EMS are then further classified by SCLpred-MEM. This predictor adapts homology reduction protocol for strict homology reduction, combined with a novel method to encode the evolutionary information of a protein. SCLpred-MEM works by generating alignments of Multiple Homologous Sequences (MSA) by iterating PSI-BLAST [17]. Predictions are made by incorporating information from membrane proteins. It is powered by an ensemble of Deep N-to-1 Convolutional Neural Networks.

mGOF-loc [18] is a multi-label ensemble learning method for predicting protein localization based on the rich sequence evolutionary information, i.e., consensus sequences. The consensus sequences are used for feature vector and ensemble classifiers to predict which protein subcellular location types belong to a particular query sequence. This predictor takes evolutionary features as a discriminating power for protein subcellular localization prediction. On the other hand, LocTree2 [19] and LocTree3 [20] are frameworks to predict localization based on a hierarchical system of support vector machines that imitates the cascading mechanism of cellular sorting. However, LocTree3 also uses the addition of homology-based inference (if available) to attain better prediction accuracy in comparison with LocTree2 [19]. YLoc+ [21] uses multiple locations using biological features (e.g., sorting signals, PROSITE patterns) in order to predict multiple locations.

PBLoc [22] is another predictor based on a feed-forward neural network (FNN) and Bidirectional Gated Recurrent Unit (BiGRU) along with a pre-trained protein model called ProtBert (Product Data Classification with Fine-tuning BERT Model). This predictor is designed based on sequence information only, and to capture sequence features accurately, an attention mechanism is used. An attention mechanism can be defined as a function that allows the model to focus on important information only, e.g., finding amino acids with higher attention weights, or focusing on attention weights of each function considered. Similarly, MPSLP [23] also uses a Self-Attention Mechanism with Deep Convolutional Neural Networks. This predictor integrates features through amino acid index distribution and physicochemical properties. It is then subjected to the use of deep convolutional neural networks for high-dimensional feature extraction. MPSLP uses an attention mechanism in order to ensure that the predictor is well-inclined towards positive data and to tackle over-sampling and under-sampling as well.

There is a growing interest in hybrid protein subcellular localization predictors that leverage multiple computational techniques to enhance prediction accuracy and reliability. One such example is SubCons [24], which uses a Random Forest Classifier (RFS) for combining four predictors together, i.e., MultiLoc2 [25], SherLoc2 [26], CELLO2.5 [5], and LocTree2 [19]. SubCons combines various features from each predictor to create a better-performing prediction tool that uses multiple methods. Another example is OrganelX [27], a hybrid predictor that uses two different approaches, i.e., protein sequence embedding via Unified Representation (UniRep) [28] and Sequence-to-Vector (SeqVec) [29]. SeqVec is based on context-dependent transfer-learning model ELMo, which is an auto-regressive model. Also, OrganelX hosts two existing algorithms, sub-peroxisomal (In-Pero) [30] and sub-mitochondrial (In-Mito) [30]. These two predicting algorithms predict peroxisomal and mitochondrial proteins on behalf of OrganelX.

## 3. Methodology

### 3.1. Approach

We introduce an ab initio protein subcellular location predictor powered by an ensemble of Deep N-to-1 Convolutional Neural Networks in eukaryotic organisms. SCLpred-ECL is trained on a recent UniProt Knowledge-base (UniProtKB/Swiss-Prot) release 2019_06 [31] and the results were measured in 5-fold cross-validation. Our system predicts eukaryotic proteins based on eight classes (Other, Cytoplasm, Golgi apparatus, Membrane, Mitochondrion, Nucleus, Plastid, and Secreted). We employed a novel homology reduction protocol for stricter homology reduction and used an in-lab encoding scheme that has led to significant performance improvements in similar predictive tasks.

We call the model that we describe in this work N-to-1 Neural Network or N1-NN. The model is based on a framework to design Neural Networks for structured data. The model aims to map a sequence of variable length N into a single property or fixed-width array of properties. Other models transform/compress the sequence into a fixed number of descriptors (or into descriptors of pairwise relations between sequences) beforehand, and they then map these descriptors into the property of interest. In N1-NN, instead, we do not compress all the information of a sequence into a handful of predefined features (e.g., k-mer frequencies, sequence length, etc.). Rather, we decide beforehand only how many features we want to compress a sequence into.

Unlike standard CNN, all convolutional kernels and the final fully connected networks are implemented by feed-forward neural networks with different sizes of hidden layers. For example, each convolutional kernel has two layers and can be seen as a proper (1-layered) kernel followed by a non-linearity, followed by a further kernel of size 1, and a further non-linearity.

### 3.2. Datasets: Materials and Methods

**Figure 1:**
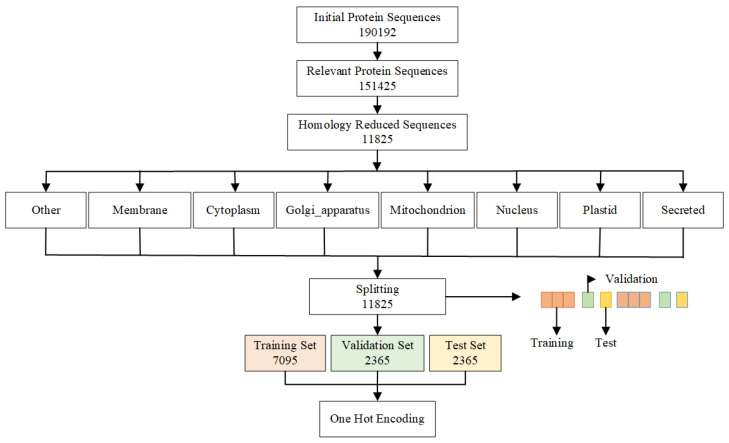
The redundancy reduction process flow from initial protein sequences to training, validation and test set splitting.

The data are derived from the recent UniProt knowledge-base release 2019 [31]. UniProt is a freely accessible database of proteins and their annotations equipped with recent and experimentally derived sequences. As illustrated in the dataset preparation timeline in Figure 1, the initial version of the dataset had 190,192 protein sequences that were theoretically annotated for subcellular localization. However, in some of these proteins, the location field was in fact empty, and ultimately only 155,143 are annotated with valid subcellular localization. These numbers are further reduced to 151,425 protein sequences as protein sequences of length greater than or equal to 30 amino acids are selected. Shorter ones are generally considered “peptides” rather than proteins and tend to follow different organizing/structuring rules.

The dataset has then been reduced to 11,825 after homology (similarity) reduction. Homology reduction is a process of removing proteins that are similar to each other beyond a certain similarity threshold. Homology reduction is necessary for better experimental accuracy. Models tend to over-fit in the presence of similar sequences and, especially, results are difficult to gauge in the absence of strict redundancy reduction between training and testing examples. The initial set of proteins is redundancy reduced using an expectation (e-value) threshold of 0.001, that is, no two sequences should be similar to each other at an expectation of 0.001 or smaller. Similarity has been assessed by BLAST alignment [17], i.e., every single protein has its similarity checked against the rest of all the proteins present in the set and all those that are more similar to it than the threshold are removed from the set before proceeding to the next protein. This redundancy reduction also automatically takes care of removing potential exact duplicates (identical sequences) in the set.

The reduced dataset of a final size of 11,825 examples is separated into eight distinct classes, each corresponding to the possible target label: Other, Cytoplasm, Golgi apparatus, Membrane, Mitochondrion, Nucleus, Plastid, and Secreted. Every protein is related to only one of the eight classes and proteins with two or multiple classes are not considered in our data. The dataset is divided with a ratio of 3:1:1 into three sets, i.e., the training set (7095), test set (2365), and validation set (2365) proteins. To ensure that all of the classes receive equal representations in all stages of model training and to avoid potential listing biases in the initial files, the sets are split in an interleaved fashion, i.e., the first three proteins are moved to the training set, the next one (fourth) is shifted to the test set, next (fifth one) is moved to the validation set, and then the cycle restarts and continues until all the 11,825 proteins are divided fully. In Table 1, data stats are shown for class sizes. Class Secreted contains the highest number of protein sequences (2392), followed by Membrane class (2372) and Nucleus (2178). The smallest class is Golgi apparatus (93), followed by Plastid (743) and Mitochondrion (784).

### 3.3. Alignments and Data Encoding

The sequences are one-hot encoded. That means that all the characters are encoded into numerical representation because of the fact that characters are unwieldy for machine learning models. One-hot encoding is preferable and easy for non-numerical data and is agnostic to the different potential similarities between characters. Each amino acid in a protein sequence is assigned a binary value ranging from 1 to 21 bits. However, one-hot encoding is memory-inefficient, as it tends to produce sparse matrices. This factor is not a particular challenge in terms of memory requirements in our case, as the number of unique amino acids is 21, i.e., each amino acid requires 21 bits only, but it does impose constraints on the size of the models themselves that have to be considered when designing their architecture.

We generated alignments of multiple homologous sequences (MSA) for all datasets by iterating PSI-BLAST [32] with an e-value of 0.001 against the latest version of UniRef90 [31]. In other words, evolutionary information in the form of alignments of multiple homologous (but unannotated) sequences is generated. These alignments are encoded into MSA profiles by calculating frequencies of residues and gaps. The frequencies of the amino acids present in the original sequence were then ‘clipped’ to 1, where each amino acid is represented by a vector of 22 frequencies of an amino acid type from the list of homologous sequences [33].

### 3.4. Predictive Architecture

**Figure 2:**
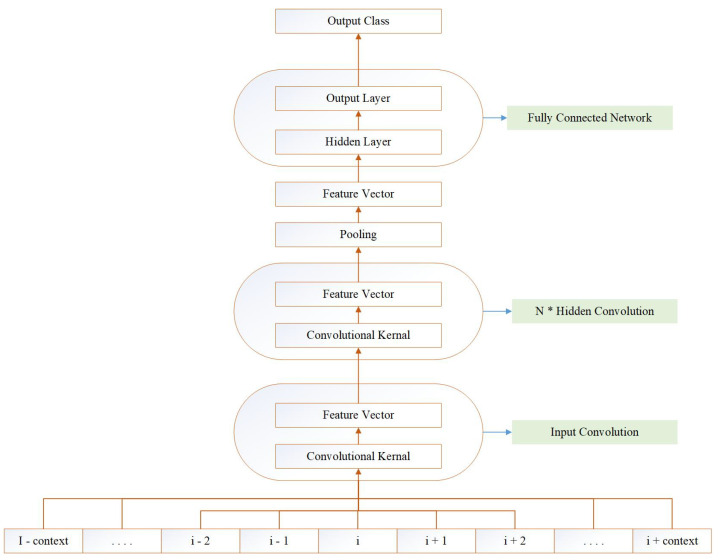
Predictive model: deep N-to-1 convolutional architecture.

We tested configurations of Deep N-to-1 Convolutional Neural Networks of various depths and widths during preliminary experiments. Deep N-to-1 Convolutional Neural Networks are composed of an input kernel mapping a window of amino acids into a feature vector followed by a stack of hidden convolutional kernels followed by an average pooling unit over the whole sequence, and a final fully connected network [34]. A complete visualization of the predictive model is given in Figure 2.

In Figure 2, the N-to-1 Neural Network, or N1-NN, is illustrated. The model is based on our framework to design Neural Networks for structured data [35,36].

The input kernel learns a non-linear function *I* from a window of amino acids $\hat{ic_{i}}$ at position *i* and predicts an intermediate state vector $\hat{is_{i}}$ at position i.

$$\hat{is_{i}}=I\left(\hat{ic_{i}}\right)$$

$$\hat{ic_{i}}=\left(i-c,...i,...,i+c\right)$$

Each hidden convolutional kernel learns a non-linear function $H^{k}$ at hidden layer *k* from a window of intermediate states $\hat{hc_{j}^{k}}$ at position *j* and predicts an intermediate state vector hsk i at position *i*.

$$hs_{i}^{k}=H^{k}\left(\hat{hc_{j}^{k}}\right)$$

$$\hat{hc_{j}^{k}}=\left(j-\gamma ,...j,...,j+\gamma \right)$$

The output vectors $\hat{hs_{p}^{l}}$ of the last hidden kernel at each position *p* are averaged element-wise into a single vector $\hat{v}$. A fully connected network predicts the final subcellular location class of each protein from the final vector $\hat{v}$. The fully connected network learns a non-linear function *O*.


$$\mathrm{cls}=O\hat{\left(v\right)}$$

In reference to the predictive model, we do not directly deal with ambiguous class assignments, e.g., proteins that might be located in multiple locations. By focusing on well-defined localization patterns, the model can achieve higher prediction accuracy for most proteins. However, proteins with multiple localizations or dynamic localization patterns present a challenge due to the lack of clear categorization. The limitation of not directly handling ambiguous class assignments, such as proteins that may be located in multiple subcellular locations, is grounded in the need for clear classification boundaries within subcellular localization prediction models.

### 3.5. Training and Ensembling

**Figure 3:**
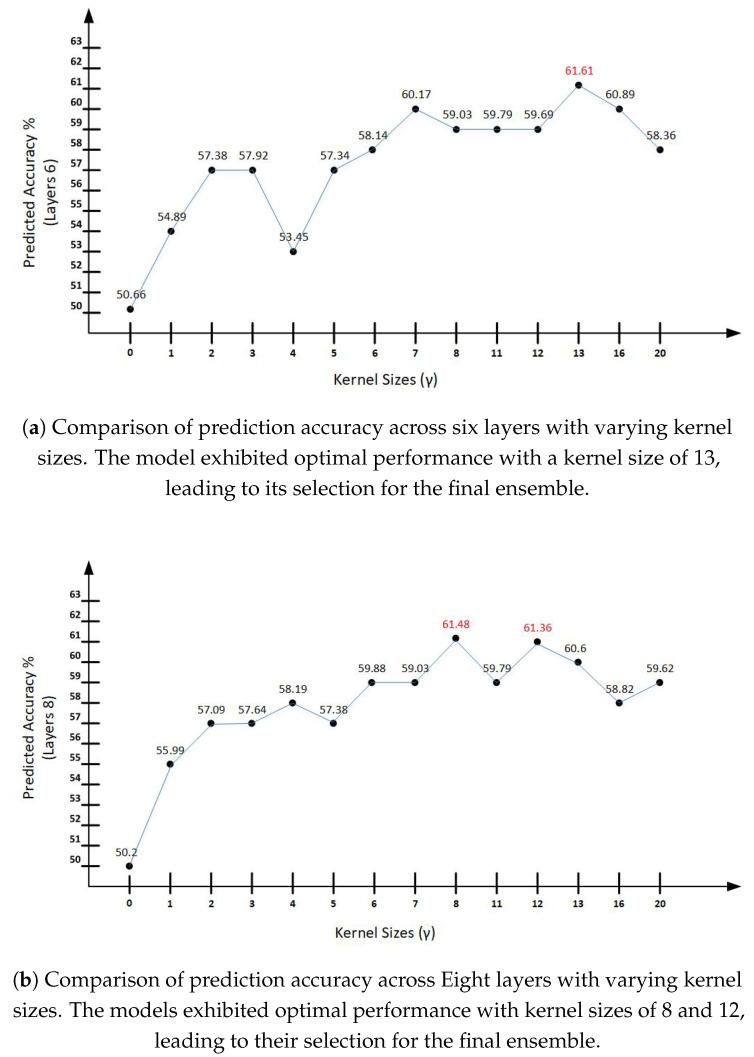

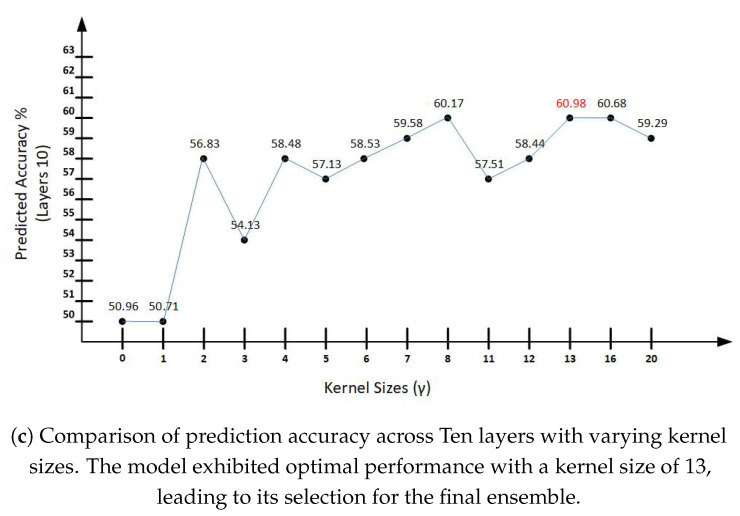
Prediction accuracies with respect to various network layers and kernel sizes.

The models are trained and tuned with the use of train, test, and validation datasets. Training proceeds by stochastic gradient descent on the relative entropy of the output vs. the target. Different trainings are conducted in a preliminary stage, searching for good sets of hyperparameters. Models are trained through 10 epochs (the whole training set is covered in each epoch), then tested on a validation set, while the ongoing error descent and other results are saved in a log file, with model parameters also saved separately for future use. This process repeats 10 times per epoch for a total of 5000 epochs. The validation set is used to steer the training/select models and gauge over-fitting, i.e., for hyper-parameter selection. In our case of 5000 epochs, this strategy produces 500 models that are related, but effectively different. Out of these 500 models, the six best-performing models on the validation set are selected. Figure 3 illustrates the best-performing instances, i.e., the ones that achieved higher prediction accuracies and are marked in red are selected as the six best-performing models. The number of epochs is a hyper-parameter, so it may be changed to a different value if these preliminary tests suggest that there is a better choice, e.g., that training tends to end before the completion of the full 5000 epochs.

### 3.6. Evaluating Performance

To measure performance for a given class *i*, we use:

$$Spec=\frac{TP}{TP+FP}$$

$$Sens=\frac{TP}{TP+FN}$$

$$Acc=\frac{TP+TN}{TP+TN+FP+FN}$$

$$MCC=\frac{TP\times TN-FP\times FN}{\sqrt{\left(TP+FP)(TP+FN)(TN+FP)(TN+FN\right)}}$$

$$F1\;Score=\frac{2\times Precision\times Sensitivity}{\;Precision+Sensitivity}$$

where:

⇒ True positives (TP): the number of sequences predicted in the membrane class that are observed in that class.
⇒ False positives (FP): the number of sequences predicted in the membrane class that are not observed in that class.
⇒ True negatives (TN): the number of sequences predicted not to be in the membrane class that are not observed in that class.
⇒ False negatives (FN): the number of sequences predicted not to be in the membrane class that are observed in that class.

For the abbreviations used in formulas for evaluating performance, (MCC) is Matthew’s correlation coefficient, (ACC) is Accuracy, (Spec) is Specificity, and (Sens) is Sensitivity. Also, the F1 score measures the predictive performance. We chose MCC because it is a widely used metric for evaluating how well binary classification models perform. It looks at factors like true positives, true negatives, false positives, and false negatives to give a fair assessment of how accurately the model classifies data. Detailed values are given in Table 2 and they are discussed in Section 5, respectively. And to evaluate the performance (*Q*) of SCLpred-ECL, the following equation is used:

$$Q=\frac{\sum_{i}Z_{ii}}{\sum_{ij}Z_{ij}}$$

where:

*Q* is performance of SCLpred-ECL;
True positives (TP): $z_{ii}$;
False positives (FP): $\sum j\neq i\;Z_{ji}$;
True negatives (TN): $\sum v\neq i\sum j\neq i\;Z_{jv}$;
False negatives (FN): $\sum j\neq i\;Z_{ij}$.

**Table 2:** Performance statistics of SCLpred-ECL for protein subcellular localization prediction across eight classes.

Subcellular	MCC	Accuracy	Specificity	Sensitivity	F1 Score
Other	0.10	26.74%	86.99%	26.74%	17.89%
Membrane	0.72	71.67%	96.02%	71.67%	77.89%
Cytoplasm	0.40	44.21%	93.34%	44.21%	47.59%
Golgi Apparatus	0.19	1.35%	92.31%	26.67%	27.00%
Mitochondrion	0.48	53.33%	97.43%	53.33%	50.45%
Nucleus	0.58	55.68%	95.66%	55.68%	65.19%
Plastid	0.50	57.50%	97.12%	57.50%	52.47%
Secreted	0.80	78.98%	97.53%	78.98%	84.05%

## 4. Results and Discussion

It should be noted that in our implementation, unlike standard CNN, all convolutional kernels and the final fully connected networks are implemented by feed-forward neural networks with two hidden layers. That is, each convolutional kernel has two layers, and can be seen as a proper (1-layered) kernel followed by a non-linearity, followed by a further kernel of size 1 and a further non-linearity. In all cases, we use sigmoidal non-linearities on the model’s internal units, rather than rectified linear units. We found both the use of deeper convolutional stages and sigmoidal units to be beneficial in preliminary tests. The resulting architecture has a minimum of three internal (hidden) layers when no hidden-to-hidden convolutional kernel is present, while an architecture with k hidden-to-hidden kernels contains 3 + 2*k* hidden layers in total.

As illustrated in Figure 3, we tried different sizes of kernels, i.e., context layer or semi-context of inputs declared as gamma (γ) for hidden layers, i.e., taking inputs by looking at the size of context layers for hidden-to-hidden networks. In this case, the number of inputs by the kernels is 2×γ+1. For the proposed model, various (γ) sizes are trained, tested, and validated to check the applicability of bigger data sizes and a larger number of classes for better prediction accuracies. We tried a value of 0 to 20 (γ) and the prediction accuracies displayed different patterns on different sizes. The prediction accuracies at (γ) 8, 12, and 13 are proven to be effective.

In Figure 3, predicted accuracies show varying trends based on kernel sizes (γ). Kernel size 0 indicates 50.66% accuracy, which is the least predicted accuracy shown at all layers. Kernel size 0 shows the same subcellular prediction accuracies irrespective of the layer depth, i.e., 50.66%, 50.2%, and 50.96%. However, prediction accuracies started showing differences in percentage from kernel size 1. It shows better results at layer 8 with 55.99%, while it shows no improvement at layer 10, i.e., sizes 0 and 1 are almost on the same predicted accuracy ratio and it is lower than layers 6 and 8. Prediction accuracies show a better trend from kernel size 8 in all deeper layers considered, whereas layer 10 started becoming slightly better a bit earlier, i.e., at kernel size 8. As per the trend line, the best-predicted accuracy is attained at kernel size 13 at layer 6, whereas kernel size 8 is better-performing among all sizes at layer size 5. At layer 10, kernel size 13 is the winning size. Apart from the most accurate values of subcellular localization prediction accuracies, kernel sizes 7, 8, 12, and 13 perform better comparatively on all layers.

When tested in a 5-fold cross-validation on the training set of 11,825 protein sequences while predicting sequences into eight classes (Cytoplasm, Golgi apparatus, Membrane, Mitochondrion, Nucleus, Plastid, Secreted and Other), the number of correctly predicted sequences by SCLpred-ECL is 63% for training, independent test, and validation sets. The number of correctly predicted sequences is 63% for training and independent test and validation sets. In order to obtain 63% accuracy, SCLpred-ECL is extensively tested for hyper-parameter and model selection. Hyper-parameter tuning is adopted through a grid search approach for fine-tuning, carried out on all datasets under consideration.

In Table 2, a detailed performance evaluation is given based on MCC (Matthew’s correlation coefficient), Accuracy (Acc) achieved, Specificity (Spec), Sensitivity (Sens), and F1 score calculated based on Section 3.5, evaluating performance measurements. Also, the detailed results are analyzed and discussed in Section 5.

In order to achieve a higher stability and greater generalization capability for the prediction, the six best-performing models from winning kernel sizes of all three layers considered are selected, i.e., kernel size 13 from layer 6, kernel sizes 8 and 12 from layer 8, and size 13 from layer 10. These models are considered based on their performance on their respective validation sets. These best-performing six models from four sizes (24 in total) were assembled together to gauge the performance against the respective test sets. The final predictor contains an ensemble of 24 models selected across different kernel sizes on the basis of the six best-performing models per four selected kernel sizes. This 24-model ensemble rectifies the performance fluctuation for unseen proteins and stabilizes the system for a better estimation of performance. It is important to note that input amino acid window sizes of 1 to 31 were tested and the generalization capability of the model deteriorated significantly after 31 amino acids. The models performed best with two hidden convolutional kernels, each containing an input window size of 11 to 31 with a learning rate that typically ranges from 0.01 to 0.02.

An extensive hyperparameter search is essential to optimize the model for higher generalization capacity. We adopted a grid search approach for hyper-parameter fine-tuning carried out on the datasets. Hyper-parameters used are the number of hidden-to-hidden convolutional stages, with a number of hidden units in the hidden-to-hidden convolutional stages. They also include the number of units in the output of the hidden-to-hidden convolutional stages and the number of hidden units in the input-to-hidden convolutional stage. Hyper-parameter fine-tuning also includes a number of outputs of the input-to-hidden convolutional stage. The width of hidden-to-hidden convolutional stages in terms of the number of hidden units and outputs of the hidden-to-hidden convolutional stages are currently kept the same for all models. Semi-context of inputs (kernel sizes) are the parameters that are experimented with to assess if deeper networks lead to better predictions (refer to Figure 3).

Initially, an extensive hyper-parameter search was run using the first fold of the dataset to find the optimal model (model selection). The hyper-parameters were tuned based on the performance of the validation dataset. The final model contains an input kernel, a hidden kernel, an average pooling unit and a fully connected network. Finally, the fully connected network reads the vector generated by the pooling unit and predicts the final results.

We chose YLoc+ [21], DeepLoc 1.0 DeepLoc 1.0 [6], and DeepLoc 2.0 [14] tools for comparison. Tools considered for comparison are recent and state-of-the-art tools. These tools have public web servers and are easily available for local implementations. Since the outputs are different for each of the methods, we map the locations to the eight classes used in this work. The compared tools do not consider Plastid and Secreted, so we are comparing the rest of the six classes in Table 3, which significantly shows that our tool SCLpred-ECL outperforms them in classes Cytoplasm, Golgi apparatus, Membrane, Mitochondrion and Nucleus. Nucleus achieved MCC of 0.58, Cytoplasm 0.40, and Mitochondrion 0.48. Also, the Cell membrane’s MCC is 0.72 and the Golgi apparatus is at 0.19. However, Plastid shows better accuracy in Deeploc 2.0 (0.90) followed by Yloc+ (0.72) and DeepLoc 1.0 (0.69), respectively.

Summarizing all discussion and results, SCLpred-ECL offers a novel and advanced approach to protein subcellular localization prediction by considering diverse classes, incorporating deeper network layers, achieving higher prediction accuracy, and outperforming existing predictors. These contributions significantly contribute to the advancement of knowledge and capabilities in the field of bioinformatics and molecular biology.

## 5. Conclusions

We have developed a subcellular localization predictor for eukaryotic protein sequences which predicts into eight classes (Cytoplasm, Golgi apparatus, Membrane, Mitochondrion, Nucleus, Plastid, Secreted, and Other) based on an N-to-1 neural network architecture (N1-NN). We have trained SCLpred-ECL in a 5-fold cross-validation on large non-redundant subsets of annotated proteins from the UniProt database 2019. We have explored the possibility of using a subcellular localization predictor trained on a diverse set of eukaryotic sequences to predict the localization of proteins with a considerably large number of classes.

The subcellular localization prediction tool named SCLpred-ECL is proven to be better at predicting eight classes (Other, Cytoplasm, Golgi apparatus, Membrane, Mitochondrion, Nucleus, Plastid, and Secreted) with an overall prediction accuracy of 63%. Our proposed tool has outperformed six subcellular locations (Cytoplasm, Golgi apparatus, Membrane, Mitochondrion and Nucleus) in comparison with YLoc+, DeepLoc 1.0, and DeepLoc 2.0 tools. Also, SCLpred-ECL is extensively trained and tested on deeper layers, stricter redundancy, and a diverse set of classes to make a state-of-the-art addition in the ab initio category of predictors.

As the amount of sequence information generated by experimental methods keeps expanding at an ever-increasing pace, it is crucial to develop and make available faster and more accurate computational methods if this abundance of sequence data is to be fully exploited. The proposed subcellular localization prediction is a step towards bridging the gap between a protein sequence and the protein’s function and can provide information about potential protein–protein interactions and insight into possible drug targets and disease processes.

Overall, while ab initio-based protein subcellular localization prediction tools offer valuable insights into the localization of proteins, they have inherent limitations that need to be considered, for example, limited prediction accuracy, higher dependency on training data, and difficulty in predicting membrane proteins. As ab initio methods often rely solely on amino acid sequence information without considering additional features, they struggle to attain higher prediction accuracy. We tried to overcome this factor with the diverse number of classes. Also, membrane proteins present unique challenges for subcellular localization prediction due to their complex structure and diverse functions. Ab initio methods may struggle to accurately predict the subcellular localization of membrane proteins. SCLpred-ECL achieved 63 % prediction accuracy to overcome this factor.

In future, we are planning to expand the scope of SCLpred-ECL in terms of broadening the scope of the prediction tool to cover a broader range of organisms, subcellular compartments, or localization patterns. Also, the plan to further improve the accuracy of prediction tools by incorporating additional features or data sources, optimizing algorithms, or refining machine learning models is another future milestone.

## Figures and Tables

**Table 1:** Data statistics of protein sequences across eight classes in training, test, and validation sets.

Class Name	Training	Test	Validation	Total
Other	964	318	537	1819
Membrane	1543	537	292	2372
Cytoplasm	922	324	344	1590
Golgi Apparatus	53	18	22	93
Mitochondrion	534	117	133	784
Nucleus	1307	430	441	2178
Plastid	467	140	136	743
Secreted	1451	481	460	2392

**Table 3:** Comparison of SCLpred-ECL performance with other state-of-the-art recent methods.

Number of Predicted Locations	MCC from YLoc+	MCC from DeepLoc 2.0	MCC from DeepLoc 1.0	MCC from SCLpred-ECL
Nucleus	0.42	0.41	0.28	0.58
Cytoplasm	0.38	0.29	0.23	0.40
Mitochondrion	0.47	0.60	0.39	0.48
Cell membrane	0.44	0.34	0.23	0.72
Golgi Apparatus	0.11	0.17	0.10	0.19
Plastid	0.72	0.90	0.69	0.50

## Data Availability

Data are available on: http://distilldeep.ucd.ie/SCL8/, accessed on 2 May 2024.
